# Catheter-Directed Interventions for the Treatment of Lower Extremity Deep Vein Thrombosis

**DOI:** 10.3390/life12121984

**Published:** 2022-11-27

**Authors:** Kajol J. Shah, Trisha L. Roy

**Affiliations:** 1 School of Engineering Medicine, Texas A&M University, Houston, TX 77030, USA; 2 Department of Vascular Surgery, DeBakey Heart and Vascular Center, Houston Methodist Hospital, Houston, TX 77030, USA

**Keywords:** deep vein thrombosis (DVT), thrombectomy, thrombolytic therapy, anti-thrombotic therapy, endovascular therapy

## Abstract

Lower extremity deep vein thrombosis (DVT) leads to significant morbidity including pain, swelling, and difficulty walking in the affected limb. If left untreated, DVT increases the risk of pulmonary embolism (PE), recurrent venous thromboembolism (VTE), and post thrombotic syndrome (PTS). The objective of this review was to identify catheter-directed interventions and their success rates for the treatment of lower extremity DVT. A comprehensive search of current and emerging catheter-directed interventions for lower extremity DVT treatment was conducted in PubMed and Google Scholar. Clinical trials, retrospective and prospective observational studies, and case reports were identified to classify percutaneous mechanical thrombectomy (PMT), catheter-directed thrombolysis (CDT), and pharmacomechanical CDT (PCDT) devices based on their mechanism of action and indication of use. Catheter-directed interventions such as PMT, CDT, and PCDT offer an alternative therapeutic strategy for DVT management, particularly in patients with limb-threatening conditions and absolute contraindications to anticoagulants. Currently, there are limited guidelines for the use of mechanical and pharmacomechanical devices because of the lack of clinical evidence available for their use in treatment. Future studies are required to determine the short and long-term effects of using catheter-directed interventions as well as their effectiveness in treating acute versus subacute and chronic DVT.

## 1. Introduction

Deep vein thrombosis (DVT) is a condition involving the occlusion of deep veins of the extremities, and often in conjunction with pulmonary embolisms (PE), affects approximately 900,000 individuals in the United States every year [1]. Venous wall injury, stasis of blood flow, and hypercoagulability increase susceptibility to DVT, particularly in the lower leg, thigh, or pelvis [2,3]. If left untreated, lower extremity DVT can increase in size and lead to further complications including post-thrombotic syndrome (PTS) and increased risk of PE [4,5,6].

Higher rates of lower extremity DVT have been evident in Coronavirus Disease 2019 (COVID-19) patients, particularly in those who were critically ill or receiving treatment in the intensive care unit [7,8,9,10]. Infection severity and inflammation as well as reduced mobility due to bed rest, use of mechanical ventilators, and venous catheterization are all possible contributory factors to the development of prothrombotic abnormalities [7,9,11,12]. Although a greater incidence of DVT has been reported in severe COVID-19 cases, DVT development was also observed in asymptomatic patients despite administration of thromboprophylaxis [13,14,15].These studies suggest the need for careful evaluation and management of patients with COVID-19 and their risk of DVT. 

The increase in DVT cases over the last few years along with thrombus complexities in different patient groups warrants individualized intervention and management of DVT. This article will identify current therapeutic strategies and technologies to combat lower extremity DVT.

## 2. Systemic Anticoagulation

Anticoagulants are considered the standard of care for treating DVT and assist in preventing thrombus growth [16,17,18]. Due to the variability in thrombus age and composition, however, they are ineffective for complete clot dissolution [19,20]. Anticoagulation can pose significant systemic bleeding risks, and residual thrombi can increase the risk of the recurrent DVT [20,21,22]. Furthermore, anticoagulant therapy is not always effective in preventing chronic complications of acute DVTs such as valvular insufficiency and PTS [23,24,25]. PTS impacts quality of life and induces significant clinical burden on patients including pain, swelling, cramping, skin discoloration, and ulcers in the affected leg [26,27].

The risk of major bleeding complications from prolonged anticoagulation should be considered in the decision criteria for patients over the age of 65, with a history of bleeding disorders, recent surgery, frequent falls, as well as those with comorbid conditions such as cancer, renal insufficiency, and impaired hepatic function [28,29,30,31]. Anticoagulants are also known to cause differences in bleeding complications between genders [32,33]. In a meta-analysis comparing the risk factors associated with Direct Oral Anticoagulants (DOAC), females were likely to have higher incidences of major and clinically relevant non-major bleeding complications than males when treated with DOACs for venous thromboembolism (VTE) [32].

Furthermore, anticoagulants such as warfarin require frequent monitoring and dose adjustment, take time in reaching target levels in the body, and can have adverse interactions with certain foods and medications [34,35]. DOACs used for DVT treatment do not require frequent monitoring; however, they are shorter acting and lack of adherence to medication regime can result in greater risk of recurrent thrombosis [34,36]. 

## 3. Catheter-Directed Intervention

**Figure 1:**
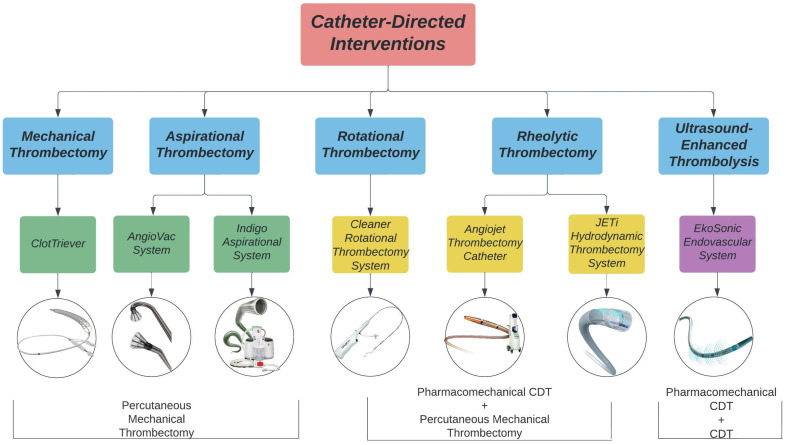
Catheter-directed interventions for lower extremity DVT treatment. Images acquired from Inari Medical, AngioDynamics, Penumbra Inc., Argon Medical, Abbott, and Boston Scientific.

Catheter-directed interventions, including percutaneous mechanical thrombectomy (PMT), catheter-directed thrombolysis (CDT), and pharmacomechanical CDT (PCDT), have been used as alternatives or adjunctive strategies of systemic anticoagulation for the treatment of DVT. The goal of these interventions is to reduce clot burden, alleviate acute symptoms, restore venous flow, and retain valvular functionality to prevent PTS in the future [37,38,39]. A summary of available catheter-directed interventions is presented in Figure 1. 

PMT devices are designed to mechanically break apart and remove thrombi from the deep veins without the use of thrombolytics. PMT devices are categorized into rotational, aspirational, rheolytic, and mechanical fragmentation devices. Rotational thrombectomy devices utilize a sinusoidal wire to spin and macerate thrombi while collecting the fragments in a built-in or separate aspiration system. Aspirational thrombectomy devices generate negative pressure to suction clots from the venous system. Rheolytic thrombectomy devices inject high pressure saline jets to break apart thrombi and create a low-pressure zone to vacuum the fragments into the catheter. Mechanical fragmentation devices manually core and separate thrombi from the venous wall. 

CDT procedures allow for infusion of lytic drugs to the thrombus site through side ports within the catheter. The Ekosonic Endovascular system can act as a CDT device alone or as a PCDT device by combining the use of thrombolytics and ultrasound to remove thrombi. Ultrasound-enhanced devices use high-frequency, low energy ultrasound waves to penetrate and dissolve clots. Rheolytic devices such as the Angiojet and JETi System and rotational devices such as the Cleaner system are additionally categorized as PCDT devices as they can infuse thrombolytics to the site of the thrombus.

## 4. Rotational Thrombectomy

### Cleaner Rotational Thrombectomy System

The Cleaner XT^TM^ (6 Fr) and Cleaner 15^TM^ (7 Fr) Rotational Thrombectomy System (Argon Medical Devices; Plano, TX, USA) are PMT catheters used for mechanical declotting and removal of wall-adherent thrombi in peripheral vasculature. A single sinusoidal-shaped wire macerates thrombi through a rotational motion at 4000 RPM. The clot is then aspirated through an introducer sheath. The Cleaner XT^TM^ uses a 9 mm sinusoidal wire for maceration of thrombi in smaller lumens whereas the Cleaner 15^TM^ uses a 15 mm sinusoidal wire for maceration of thrombi in larger lumens. The Cleaner system also has an infusion port for the delivery of thrombolytics. 

To evaluate the effectiveness of the Cleaner thrombectomy device in DVT treatment, patients with acute or subacute lower-extremity DVT (*n* = 41) with leg swelling, pain, or symptoms of phlegmasia cerulea dolens (PCD) were recruited. With one PMT session, 97.6% of patients had greater than 50% thrombus resolution after treatment. In total, 29 of those patients had complete thrombus resolution whereas 11 patients had 50–99% resolution. Complete resolution of subacute DVTs (37.5%) was lower than acute DVTs (78.8%). One major bleeding episode occurred with no incidence of systemic bleeding complications [40]. 

In another study, there was an 87.5% success rate in complete or near complete thrombus removal as well as complete restoration of blood flow using the Cleaner System in patients with iliofemoral and/or femoropopliteal DVT (*n* = 16). The Cleaner System was not effective in two patients as they had subacute DVTs with symptoms lasting for 14 days before intervention. Although there is a risk of endothelial wall injury with rotational devices, no major vein wall perforations were indicated from the system [41].

The studies mentioned above used the mechanical and thrombolytic infusion system of the Cleaner device during treatment. To avoid micro and macro emboli, IVC filters were used. Additionally, to promote continuous restoration of venous flow, balloon angioplasty and/or stenting were selectively used. The Cleaner Rotational Thrombectomy System was effective in the treatment of acute iliofemoral and femoropopliteal DVT [40,41,42]. Long-term outcomes using the Cleaner were reported in a study by Yuksel et al. (2017), which demonstrated a 67.5% PTS-free survival rate after a mean follow-up time of 16 months [42].

## 5. Aspiration Thrombectomy 

### 5.1. Indigo Aspiration System

The Indigo Aspiration System (3.4–12 Fr) (Penumbra, Inc.; Alameda, CA, USA) is an aspiration mechanical thrombectomy system composed of a catheter, separator, and vacuum pump. The Penumbra ENGINE delivers continuous suction for thrombus removal. The Indigo Separator^TM^ aids in thrombus fragmentation and cleaning of the catheter. Through its dual pressure sensors, The Lightning^TM^ Intelligent Aspiration Tubing monitors blood flow in real time. This allows for the system to automatically control the degree of aspiration and reduce blood loss by providing intermittent aspiration around the venous segment and continuous aspiration near the clot. 

In a study treating 10 patients with iliofemoral or central DVT using the Indigo System, six patients had greater than 70% thrombus resolution without the use of lytic therapy. Three patients had to be treated with CDT, angioplasty, and/or stenting due to residual thrombosis [43].

Utilization of this system in another study for acute iliofemoral DVT treatment (*n* = 16) demonstrated greater than 70% thrombus resolution in all patients. There were no serious post-treatment complications such as transfusions, hematomas, or new renal insufficiency developments. Follow-up one to eight months after treatment showed venous patency in 93.8% of the patients and 85.7% were symptom free [44]. 

The Indigo Aspiration device provides a safe alternative in the treatment of acute iliofemoral and central DVT as it minimizes the aspiration of blood and reduces the need for the administration of thrombolytics. In a study by Saleem et al. (*n* = 20), 75% of patients did not require lytic delivery and 70% of patients had patency after 3–6-months post follow-up [45].

### 5.2. AngioVac System

The AngioVac System (18, 22 Fr) (AngioDynamics; Latham, NY, USA) is a vacuum-assisted thrombectomy device indicated for removing fresh, soft thrombi or emboli through a veno–venous bypass circuit. The system utilizes a suction cannula to collect clot fragments and filters the fragments through a canister before returning the blood into the reinfusion cannula. The system has a self-expanding funnel shape tip to enhance venous drainage flow and prevent clogging of the cannula. The device is available with a 20- or 180-degree angled tip to facilitate navigation through the vasculature.

In the RAPID registry analyzing 91 patients with acute and chronic caval thromboemboli, 73.6% had greater than 70% clot removal whereas 13.2% had around 51–70% clot removal after using the Angiovac system. There was one procedure-related death due to an embolism from the IVC traveling to the pulmonary arteries [46].

Four patients with iliocaval thrombosis (acute *n* = 3; chronic *n* = 1) had complete clot extraction after treatment with the AngioVac system. Two of these patients had clots extending into their IVC filter but developed no complications with thrombectomy and filter removal [47]. In another study, the AngioVac system was used as an alternative interventional strategy after unsuccessful thrombolysis through CDT (*n* = 3). The device aided in removing nearly all the clot from the femoral and iliac veins as well as removing thrombus extensions within the IVC filter. All three patients had successful clot removal and symptom relief despite having a prior history of coagulation and DVTs [48]. 

The AngioVac System also demonstrated an 88% (*n* = 8) effectiveness in treating chronic iliocaval thrombosis, which was defined by the author as having a diagnosis of DVT > 28 days [49]. Due to the large bore cannulas of the Angiovac system, however, DVT treatment is limited to the IVC and iliofemoral veins [50]. Additionally, the added rigidity of the device limits maneuverability through the venous system [48,49].

## 6. Mechanical Fragmentation

### ClotTreiver

The ClotTriever BOLD^TM^ (16 Fr) (Inari Medical; Irvine, CA, USA) is a mechanical thrombectomy device that is designed to remove large thrombi from peripheral vasculature. The system includes two elements: the ClotTriever catheter, which contains a nitinol coring element and braided collection bag, as well as the ClotTriever sheath, which contains a self-expanding nitinol funnel and a side port for aspiration. Once the guidewire is inserted beyond the clot, the ClotTriever sheath is positioned over the guidewire distal to the clot and the funnel is expanded for clot capture. The ClotTriever catheter is advanced through the sheath beyond the clot where the nitinol coring element and collection bag are deployed. As the coring element is retracted back towards the sheath, the thrombus is separated from the venous wall and captured in the collection bag. The side port can be used for aspiration of remaining clot fragments in the sheath. The device can be reinserted multiple times to allow for extensive thrombus capture. 

In one of the first published case series using the ClotTriever system (*n* = 12), 100% of patients presenting with acute iliofemoral, femoropopliteal, or iliocaval DVT had complete clot and symptom resolution. There were no postoperative complications of acute kidney disease or PE. Follow-up (mean of 4 months) with 11 patients showed that 90.9% had minimal symptoms or remained symptom free and 80% had persistent patency of the treated venous segment [51].

The ClotTreiver Outcomes (CLOUT) registry evaluated the effectiveness of the ClotTriever system in treating patients with DVT in the femoral veins, iliac veins, or IVC (*n* = 244). More than 75% thrombus removal was achieved in 90.8% of acute, 81.9% of subacute, and 83.8% of chronic DVTs, and no thrombolytics were used during the procedures [52]. A total of 99.6% of patients were treated in a single session with a median hospital stay of about 1 day. As chronicity of the clot increased, median thrombectomy time and treatment with angioplasty increased.

## 7. Ultrasound-Enhanced Thrombolysis 

### EkoSonic Endovascular System

The EkoSonic Endovascular System (EKOS Corporation, Bothell, WA, USA) is an ultrasound-enhanced device that aids in the controlled and selective infusion of thrombolytics to peripheral vasculature to dissolve thrombi. The catheter has two parts: the infusion catheter and the ultrasonic core. The infusion catheter uses an infusion pump to deliver lytic agents through its side holes and allows for the flow of saline to cool the ultrasound core while activated. The ultrasound core generates radial pressure that increases access to receptor sites for lytic agents and accelerates lytic dispersion into the thrombus.

Patients with chronic iliofemoral or femoropopliteal DVTs (*n* = 12) were treated with the EkoSonic system to restore patency of the venous segment and improve clinical symptoms that lasted for more than 28 days. Six patients had complete clot lysis (95%) and five patients had partial clot lysis (50–95%). In total, 83% of the patients showed significant or complete clinical improvement in the affected limb. There were no serious complications from the treatment and after an average of 9 months post treatment, none of the 11 successfully treated patients had developed severe PTS [53]. 

Another study recruited patients with either acute (*n* = 40) and chronic (*n* = 8) DVTs in the iliofemoral or popliteal segments. Complete lysis occurred in 79% of patients and partial lysis occurred in 21% of patients. Of the eight patients with chronic DVT, six achieved complete thrombus resolution; however, there was limited interventional differences mentioned in the study besides prolonging the administration of thrombolytics. Several patients who had a history of recurrent DVTs had greater difficulty achieving complete thrombolysis than those without recurrent DVT (*p* = 0.001). A one-year follow-up showed that six patients had developed recurrence and needed repeat thrombolysis, whereas the rest of the patients demonstrated continued patency [54].

Although the above studies demonstrate feasibility in utilizing the EKOS device in chronic and acute DVTs, larger randomized trials are needed to determine its effectiveness in treating more organized clots. Additionally, there is still a debate of whether ultrasound improves thrombus removal better than CDT alone. In the BERNUTIFUL trial, the addition of ultrasound using the Ekosonic device did not increase thrombus resolution and did not affect PTS severity in patients with acute iliofemoral DVT in comparison to CDT alone [55].

## 8. Rheolytic Thrombectomy System 

### 8.1. AngioJet Thrombectomy Catheter

The AngioJet Thrombectomy Catheter (Boston Scientific; Natick, MA, USA) delivers high pressure saline jets through the tip of the catheter. The saline jets travel backwards to create a low-pressure zone, which allows for the thrombus to be suctioned into the in-flow windows. ZelanteDVT Catheter (8 Fr) is designed to treat upper and lower peripheral veins with a diameter of ≥6 mm. The Solent Omni and Proxi Catheters (6 Fr) are used for treating veins that are ≥3 mm in diameter. The AngioJet system also has a Power Pulse feature which allows for the infusion of thrombolytics at the site of the thrombi.

Evaluation of the PEARL registry with 329 patients undergoing lower extremity DVT treatment with the AngioJet device resulted in complete or 50–99% clot reduction in 96% of the patients. Total procedure time in 73% of patients was less than 24 h and bleeding complications occurred in 4.5% of patients, none of which were related to the Angiojet device [56]. 

In a study evaluating the effectiveness of the AngioJet system in subacute lower extremity DVT cases (onset of thrombosis between 15–75 days), 63% of patients (17/27) had clot resolution between 50–99% and 37% of patients (10/27) had less than 50% resolution. Further treatment with CDT, a stent, and angioplasty in 19 patients resulted in a total of two cases with complete clot resolution, 23 cases with a 50–99% reduction, and two with less than a 50% reduction [57]. 

The AngioJet system was also useful in clearing acute IVC filter-related thrombosis (*n* = 33). In 20 patients, the thrombus was resolved by more than 90% and in 13 patients, it was resolved by 50–90%. Three patients developed hemoglobinuria, which was alleviated after urine alkalization treatment and one patient developed recurrent thrombosis 4 months after intervention, which was resolved with CDT treatment [58]. 

In a comparison study between the Angiojet System and CDT, there were no differences in thrombus clearance, complete symptom improvement, and serious complications between both two procedures [59]. Duration of treatment, drug dose, and PTS severity scores, however, were lower with the Angiojet device. Due to high pressure saline injections, the Angiojet system has possible risk of hemoglobinuria, acute kidney injury (AKI), and bradycardia [59,60,61]. 

### 8.2. JETi Hydrodynamic Thrombectomy System

The JETi^TM^ Hydrodynamic Thrombectomy System (6F and 8F) (Abbott; Irvine, CA, USA) uses a combination of aspiration and high-pressure saline jets to suction and fragment clots from the venous system. The outer conduit allows for continuous thrombus aspiration while the inner catheter tip utilizes saline jets to break up the aspirated thrombus and reduce catheter clogging. The JETi device also has a HyperPulse^TM^ Fluid Delivery feature which allows for selective infusion and delivery of thrombolytics. 

In a study evaluating the effectiveness of the JETi Thrombectomy System in the treatment of iliocaval, iliofemoral, and femoropopliteal DVTs (*n* = 53 limbs, 47 patients), 79% of limbs had flow restoration after using the device alone (42/53) [62]. After adjunctive therapy using balloon angioplasty and stenting, 88.6% of limbs had flow restoration. Overnight thrombolysis allowed for three additional limbs to have unobstructed flow (50/53). There were no adverse events of PE, major bleeding, or death. Although patients with both acute and subacute DVTs were included in this study, a comparison between the effectiveness of the JETi system with subacute DVTs was not measured. Additionally, patients were not followed long term to assess for outcomes of recurrent VTE and PTS. 

Another retrospective study evaluated the procedural success of the JETi system in patients with iliofemoral DVT who had symptoms lasting less than 14 days (*n* = 9) [63]. In a single session, there was an average of 81% clot reduction (range: 60–100%). Five of the procedures required additional overnight CDT for greater thrombus removal, which ultimately resulted in an average of 96% clot reduction (range: 80–100%). There were no major adverse events associated with the device, however, there was one patient that had recurrent thrombosis 5 days later. 

The JETi system offers the potential to aspirate large volumes of acute DVTs in both small and large venous segments. With an internal high pressure saline jet, the JETi reduces risk of hemolysis and embolic complications [63,64]. Loss of blood volume due to aspiration, however, can be a potential complication with the JETi system, especially in anemic patients [63]. Future studies are required to determine long-term complications after using this device as well its effectiveness in subacute and chronic clots. 

## 9. Discussion

Lower extremity DVT requires careful evaluation of location, onset, symptom severity, as well as consideration of patient preference and medical history for optimal intervention. DVTs located within the popliteal, femoral, or iliac veins are considered proximal DVTs [65,66]. Isolated distal DVTs are located below the popliteal vein and do not extend into the proximal veins. Proximal DVTs have a higher embolic potential than distal DVTs, which is why management of distal DVTs is controversial and often reserved for high-risk patients [66]. Thrombus remodeling of isolated distal DVTs, however, is possible [67]. Distal DVTs can extend proximally in an ascending pattern, which can increase the risk of PE. Descending distal DVTs are less common [67].

Anticoagulants used for DVT treatment assist in reducing thrombus progression and occurrence of PE, but incomplete resolution increases the risk of recurrent VTE, valvular insufficiency, and PTS. Inferior Vena Cava (IVC) Filters are indicated as an alternative treatment modality for patients with absolute contraindications to anticoagulation but create additional complications including IVC thrombosis, arteriovenous fistulas, and have poor retrieval rates [68,69,70]. Elastic compression stockings (ECS) are used in DVT management to increase venous return as well as reduce edema and swelling post-treatment [71]. In a meta-analysis evaluating compression therapy and prevention of PTS in DVT patients, the use of compression stockings led to lower incidence of PTS [72]. Due to low quality of evidence and high heterogeneity between studies, however, the efficacy of ECS in preventing PTS is still debated [72,73]. ECS, should thus be used based on provider judgement, particularly taking into consideration patient compliance for prolonged ECS use [73]. 

CDT offers the ability to directly infuse thrombolytics to the clot site; however, long treatment time can increase risk of hemorrhage [74,75]. Due to bleeding risks, it is critical to evaluate the risk-to-benefit ratio in utilizing CDT in the treatment of DVT. The 2020 NICE guidelines recommend CDT for individuals with iliofemoral DVT who have symptoms lasting less than 14 days, have low risk of bleeding, good functional status, and a life expectancy of one or more years. 

PMT and PCDT are designed to allow for faster and more complete resolution of thrombi thereby reducing administration of thrombolytics and time required for treatment [52,76]. Due to a risk of thrombus embolization from mechanical thrombectomy, temporary prophylactic deployment of an IVC filter may be considered in patients, particularly those with proximal DVTs [50].

The open-vein hypothesis states that early removal of thrombi allows for the preservation of venous patency and valvular function which leads to a lower incidence of PTS [37]. The effectiveness of PCDT in reducing periprocedural complications and rates of PTS, however, remains controversial. The ATTRACT trial, which was the largest randomized trial evaluating catheter-directed interventions for acute proximal DVTs (*n* = 692), reported that PCDT resulted in higher risks of major bleeding and did not reduce risk of PTS in comparison to the group treated with anticoagulants alone [77]. Another meta-analysis (*n* = 1323) demonstrated that PMT ± CDT had better efficacy in thrombus removal at a lower thrombolytic dosage and procedural time than CDT alone. Additionally, PMT ± CDT resulted in low thrombosis recurrence rates, perioperative complications, and incidence of PTS [78].

Guidelines for the treatment of DVT with PMT are limited due to the lack of randomized trials evaluating the efficacy of PMT devices [79,80]. The 2019 NICE guidelines recommend using PMT for acute DVT if thrombolytics are contraindicated or as an adjunctive strategy before thrombolytics or after to remove residual thrombus [81]. The 2021 CHEST guidelines recommend utilizing catheter-directed interventions in patients with severe, limb-threatening DVT, such as in PCD or threatened venous gangrene [82]. Both the 2019 NICE and 2016 AC Forum guidelines recommend PMT use on an individual basis with careful consideration to patient age as well as length of DVT presentation and symptom severity [81,83].

The lack of specific guidelines on the use of PMT and PCDT devices could additionally be attributed to the fact that older devices such as the Trellis^TM^ Thrombectomy System and the Arrow Trerotola^TM^ Percutaneous Thrombolytic Device are no longer available, whereas newer devices such as the Wolf Thrombectomy System (Devoro Medical; Fremont, CA, USA), Bashir^TM^ Endovascular Catheter (Thrombolex Inc.; New Britain, PA, USA), Uni-Fuse^TM^ Infusion Catheter (AngioDynamics; Latham, NY, USA), and QuickClear Mechanical Thrombectomy System (Philips; San Diego, CA, USA) require time for clinician education and implementation in hospitals. Furthermore, a limited number of studies have tested PMT/PCDT devices in large patient populations and monitored the occurrence of chronic DVT complications including recurrent VTE and PTS after treatment. 

Future studies should also investigate how to accurately categorize acute versus subacute and chronic DVTs as well as what catheter-directed interventions are most appropriate for each type. In a study evaluating the chronicity of thrombi from the CLOUT registry, 49% of extracted thrombi were more chronic than that what was indicated by the duration of symptoms [52]. This discrepancy suggests the importance of pre-thrombectomy imaging in conjunction with evaluating symptom duration when determining thrombus chronicity. 

**Figure 2:**
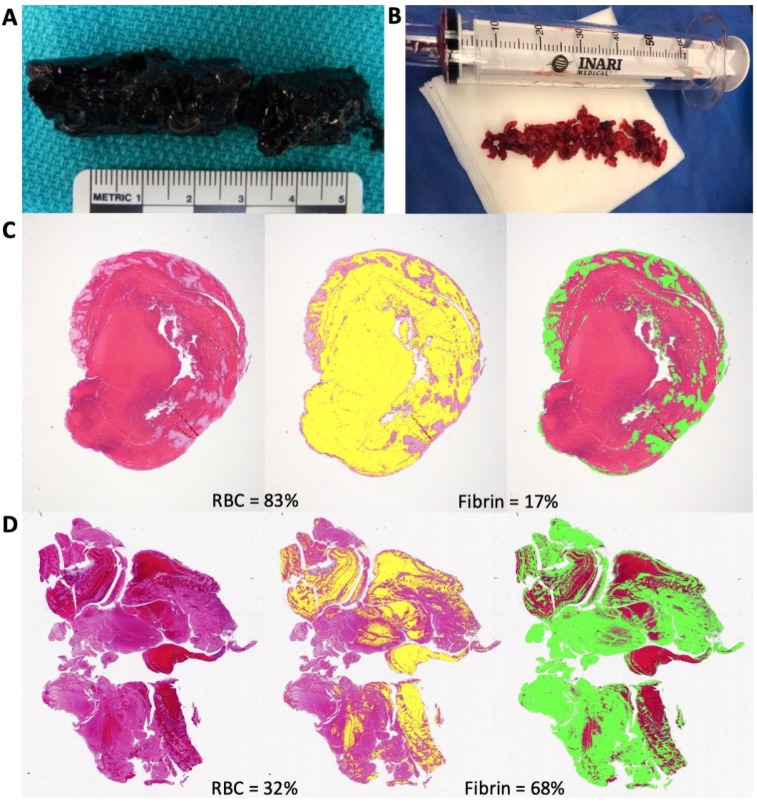
Complete thrombus removal was achieved by the ClotTriever in an acute lower extremity DVT case with a 1-day history of left leg pain (**A**). Incomplete thrombus resolution with the ClotTriever was observed in another lower extremity DVT case with a 2-day history of left leg pain (**B**). Separation of RBC from the fibrin component of the clot segments demonstrated that the more acute clot had a higher RBC concentration with majority of the fibrin localized to the edges (**C**). The more chronic clot had a higher concentration of fibrin distributed across the segment (**D**).

We also discovered a similar trend when managing two patients with DVT extending from the iliofemoral to the popliteal segments. A 48-year-old male with a 1-day history of left leg pain and no relevant past medical history had complete clot resolution and marked symptom relief after intervention with the ClotTriever (Figure 2A). A 40-year-old female who had prior COVID-19 and a 2-day history of left leg pain, in comparison, had incomplete clot resolution and minimal symptom relief even after multiple attempts with the ClotTriever (Figure 2B). Histological analysis of the retrievable clot demonstrated a greater composition of erythrocytes than fibrin along with the fibrin localized to the edges of the clot (Figure 2C). The incompletely resolved clot had a larger fraction of fibrin distributed throughout the segment with significantly lower composition of erythrocytes (Figure 2D). Although both cases appeared to be acute DVTs based on the symptom duration, characterization of both clots after the procedure demonstrated that the second patient had a more chronic clot. 

A few interventional devices such as the Angiovac, ClotTriever, Ekosonic, and Angiojet showed success in treating DVTs lasting longer than 14 days; however, these studies were limited to a small patient size [49,52,53,57]. Newer mechanical devices are being introduced into the market that are designed to target subacute and chronic clots. Wolf Thrombectomy by Devoro Medical is designed to ingest soft and organized clots; however, there are limited studies evaluating its efficacy clinically. Another promising device is the ClotHunter by Boston Scientific, which is the newest addition to the AngioJet Thrombectomy Systems. The ClotHunter is a helical shaped attachment for the ZelanteDVT device that rotates 330 degrees clockwise and counterclockwise to target wall-adherent clots. It is designed to remove more challenging acute and subacute DVTs. Bench testing by Boston Scientific with the ClotHunter and ZelanteDVT demonstrated 60–80% greater thrombus removal than with the ZelanteDVT alone. Currently, however, there is a lack of evidence supporting the efficacy of the ClotHunter clinically. Future studies will need to focus on determining which interventional devices are most appropriate for subacute and chronic clots.

In select acute DVT patients, such as those with rehabilitating symptoms, PCD, and venous gangrene, open surgical thrombectomy may be used as an alternative treatment modality to restore venous patency [84]. Open surgery may also be performed in patients with failed endovascular attempts and contraindications to thrombolytics [85,86]. Perioperative complications and the length of post-surgical recovery times, however, must be taken into consideration when choosing an open versus endovascular approach [86]. 

## 10. Conclusions

Catheter-directed interventions for the treatment of lower extremity DVT include catheter-directed thrombolysis (CDT), percutaneous mechanical thrombectomy (PMT), and pharmacomechanical CDT (PCDT). CDT allows for direct infusion of thrombolytics to the site of the clot and is recommended for patients with acute iliofemoral DVT, symptoms less than 14 days, acute limb threat, lower bleeding risk, and have higher life expectancy. 

PCDT and PMT devices reduce the thrombolytic dose, treatment time, and hospital stay. Utilization of these devices should be done on a case-by-case basis, particularly in patients with contraindications to lytic therapies and require immediate intervention. Mechanical intervention can be achieved through rotational, aspirational, mechanical fragmentation, ultrasound, and rheolytic thrombectomy to remove emboli from the venous system. 

Currently, there is a lack of evidence and detailed guidelines supporting the use of PMT and PCDT devices for DVT treatment. There is an unmet clinical need in determining which catheter-directed interventions are required for treating DVT based on thrombus location, DVT onset, symptom severity, patient age, and medical history. Future studies will need to determine the safety profile and the effectiveness of these therapies in treating acute, subacute, and chronic DVTs as well as its long-term effects of developing VTE and PTS.

## Data Availability

Not applicable.
